# The Hippocampal Barque: An Epileptiform but Non-epileptic Hippocampal Entity

**DOI:** 10.3389/fnhum.2020.00092

**Published:** 2020-03-18

**Authors:** Vasileios Kokkinos, Robert Mark Richardson, Alexandra Urban

**Affiliations:** ^1^Department of Neurosurgery, Massachusetts General Hospital, Boston, MA, United States; ^2^Harvard Medical School, Boston, MA, United States; ^3^Department of Neurology, School of Medicine, University of Pittsburgh, Pittsburgh, PA, United States; ^4^University of Pittsburgh Comprehensive Epilepsy Center, Pittsburgh, PA, United States

**Keywords:** epilepsy, epilepsy surgery, hippocampus, SEEG, 14&6/s positive spikes

The advent of stereotactic electroencephalography has provided us with the unique opportunity to record intrinsic hippocampal activity, and the potential to discriminate between normal and pathologic hippocampal discharges. Although the hippocampal spindle has been well-documented as a normal variant of archicortical activity that promotes memory consolidation (Ferrara et al., 2012), there are several uncategorized patterns of hippocampal activity that, even though they stand out of the intracranial EEG background and can incorporate paroxysmal-like morphological features, cannot be confidently categorized as interictal epileptic activity. Frauscher isolated atypical hippocampal discharges on the basis that their morphology shared a mixture of paroxysmal and oscillatory features (Frauscher et al., 2015a). Given that recordings from the human hippocampal formation are derived from invasive investigations in the context of evaluations for epilepsy surgery, there has been objective difficulty in discriminating between normal and pathologic manifestations of hippocampal activity. Both Montpaisir and Malow reported hippocampal entities that, although they manifested with the characteristic spindle oscillation morphology, were “crowned” by high amplitude spike discharges, thereby raising valid questions whether their nature was epileptic (Montplaisir et al., 1981; Malow et al., 1999).

This distinct category of atypical hippocampal activity appearing in recordings from both epileptic and non-epileptic hippocampi has been recently shown to be correlated and time-locked to the ipsilateral appearance of the 14&6/s positive spikes variant on the scalp (McLachlan and Luba, 2002; Jain et al., 2018, 2019; Kokkinos et al., 2019). This hippocampal waveform is comprised of bursts of high-amplitude negative-phase spikes, of a ramping up—ramping down amplitude profile, often overlaid on low-amplitude slow waves (Figure 1). The 14/s counterpart of the ctenoid variant is time-locked to sharp negative hippocampal spikes (Figure 1A, left), while the 6/s counterpart is time-locked to sharp negative spikes riding low-amplitude slow waves (Figure 1A, right). Although this atypical hippocampal waveform shares the same sigma frequency band (12–16 Hz) with spindles, they are clearly distinct both in terms of morphological features and temporal correlation/level of synchrony to the respective scalp EEG waveforms (Frauscher et al., 2015b) (Figures 1B,C).

**Figure 1:**
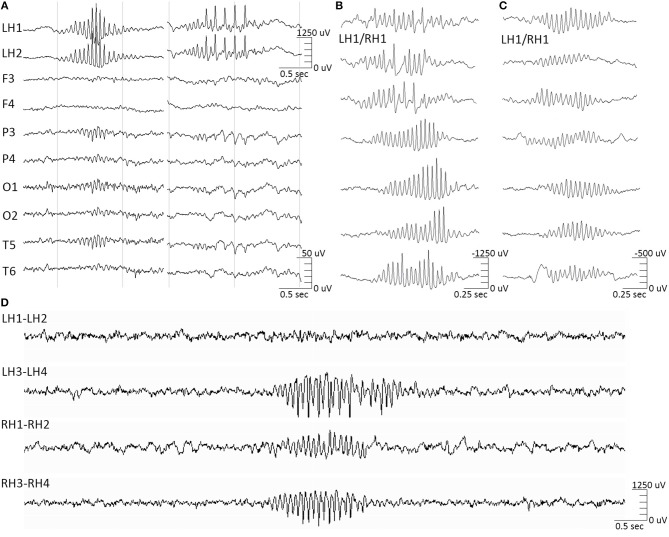
Hippocampal barques. (**A**, left) 14 Hz-only hippocampal barques correlate to the scalp 14/sec-only positive spikes variant. (**A**, right) 14&6 Hz hippocampal barques correlate to the scalp 14&6/sec positive spikes variant. The intracranial and extracranial signals are time-locked. **(B)** Featured samples of hippocampal barques, derived from various instances of the intracranial recording, highlighting their morphological variation. All samples were derived from an electrode contact implanted in non-epileptic hippocampal tissue in patients with extra-temporal focal epilepsy in which either hippocampus was not part of the seizure onset zone, either on the right or left hippocampus. Samples are not time-locked between them, but each of them was time-locked to the extracranial 14&6/sec positive spikes variant. Concurrent EEG/sEEG recordings were referenced at the midline CPz scalp electrode position. **(C)** Featured samples of hippocampal spindles for morphological comparison against the barques. These samples were also derived from the same electrode contacts as **(B)** and are not time-locked between them. Hippocampal spindles had no correlation to the extracranial 14&6/sec positive spikes variant. Note the difference in amplitude scales between the 2 entities. **(D)** Hippocampal barques recorded from a bilateral hippocampal RNS implantation (LH, left hippocampus; RH, right hippocampus).

We propose the term hippocampal “barque” for this formerly atypical hippocampal entity corresponding to the 14&6/s positive spikes variant, due to its morphological resemblance to eighteenth century sailing vessels with more than three masts (Oxford English Dictionary, 2005). For this intracranial entity we propose a term discrete from the terms used to describe the scalp manifestations (14&6/s, ctenoids), as the neural substrate generators are, respectively, discrete (archicortex vs. neocortex). The high temporal correlation of the hippocampal barques to the manifestation of a scalp EEG variant that appears invariably among normal and pathologic populations (Schwartz and Lombroso, 1968) suggests that they constitute a variant of hippocampal activity equally normal to hippocampal spindles. However, barques have an epileptiform morphology that can be easily misinterpreted as genuine interictal epileptic activity. Despite the fact that barques manifest as trains of high-voltage negative-phase spikes, thereby resembling epileptiform discharges, evidence derived from non-epileptic hippocampal tissue in patients with extra-temporal focal epilepsy, where it was shown that the hippocampus was not part of the seizure onset zone, show that they are not epileptic (Kokkinos et al., 2019). Therefore, we recommend that hippocampal barques should not be considered as markers of epileptogenicity in mesial-temporal intracranial investigations for epilepsy surgery, as well as in responsive neurostimulation (RNS) post-implantation evaluations (Figure 1D).

## Author Contributions

All authors listed have made a substantial, direct and intellectual contribution to the work, and approved it for publication.

### Conflict of Interest

The authors declare that the research was conducted in the absence of any commercial or financial relationships that could be construed as a potential conflict of interest.
